# Positive Effects of Voluntary Running on Metabolic Syndrome-Related Disorders in Non-Obese Hereditary Hypertriacylglycerolemic Rats

**DOI:** 10.1371/journal.pone.0122768

**Published:** 2015-04-01

**Authors:** Vojtěch Škop, Hana Malínská, Jaroslava Trnovská, Martina Hüttl, Monika Cahová, Agnieszka Blachnio-Zabielska, Marcin Baranowski, Martin Burian, Olena Oliyarnyk, Ludmila Kazdová

**Affiliations:** 1 Center for experimental medicine, Institute for Clinical and Experimental Medicine, Prague, Czech Republic; 2 Department of Biochemistry and Microbiology, University of Chemistry and Technology, Prague, Czech Republic; 3 Department of Physiology, Medical University of Bialystok, Bialystok, Poland; Faculty of Biology, SPAIN

## Abstract

While metabolic syndrome is often associated with obesity, 25% of humans suffering from it are not obese and the effect of physical activity remains unclear in such cases. Therefore, we used hereditary hypertriaclyglycerolemic (HHTg) rats as a unique model for studying the effect of spontaneous physical activity [voluntary running (VR)] on metabolic syndrome-related disorders, such as dyslipidemia, in non-obese subjects. Adult HHTg males were fed standard (CD) or high-sucrose (HSD) diets *ad libitum* for four weeks. Within both dietary groups, some of the rats had free access to a running wheel (CD+VR, HSD+VR), whereas the controls (CD, HSD) had no possibility of extra physical activity. At the end of the four weeks, we measured the effects of VR on various metabolic syndrome-associated parameters: (i) biochemical parameters, (ii) the content and composition of triacylglycerols (TAG), diacylglycerols (DAG), ceramides and membrane phospholipids, and (iii) substrate utilization in brown adipose tissue. In both dietary groups, VR led to various positive effects: reduced epididymal and perirenal fat depots; increased epididymal adipose tissue lipolysis; decreased amounts of serum TAG, non-esterified fatty acids and insulin; a higher insulin sensitivity index. While tissue ceramide content was not affected, decreased TAG accumulation resulted in reduced and modified liver, heart and skeletal muscle DAG. VR also had a beneficial effect on muscle membrane phospholipid composition. In addition, compared with the CD group, the CD+VR rats exhibited increased fatty acid oxidation and protein content in brown adipose tissue. Our results confirm that physical activity in a non-obese model of severe dyslipidemia has many beneficial effects and can even counteract the negative effects of sucrose consumption. Furthermore, they suggest that the mechanism by which these effects are modulated involves a combination of several positive changes in lipid metabolism.

## Introduction

A sedentary lifestyle and the increased consumption of sugar, high-fructose corn syrup and energetically-rich food have contributed to a rapid increase in the prevalence of obesity, metabolic syndrome and type 2 diabetes [1–3]. For these reasons, the positive role of physical activity on metabolism is being intensively studied. It is widely known that physical activity has a beneficial effect on reducing the metabolic dysfunctions associated with obesity [4,5]. However, 25% of humans with metabolic syndrome (diagnosed according to National Cholesterol Education Program Adult Treatment Panel III criteria) are neither overweight nor obese [6], and the effect of physical activity on metabolic syndrome-related disorders in such cases remains unclear.

Fructose overconsumption is believed to be involved in the development of many metabolic disorders, such as dyslipidemia, obesity and insulin resistance [2,7,8]. This is, at least partly, associated with the utilization of fructose in liver *de novo* lipogenesis, which results in increased liver lipid content and increased very-low-density lipoprotein (VLDL) secretion [9–11]. Via this mechanism, fructose contributes to lipid deposition in non-adipose tissue, mainly in the liver and muscles [12].

An increase in intracellular triacylglycerols (TAG) in the liver and muscles is associated with insulin resistance in these tissues [13–15]. One of the causes of insulin resistance, and thus of metabolic syndrome, is the elevated content of lipotoxic intermediates, primarily diacylglycerols and ceramides. Acting as signal molecules, they affect cellular metabolism and insulin signal transduction [16,17]. Apart from elevated amount of lipids and lipotoxic intermediates, another important factor appears to be the fatty acid composition of individual lipid classes, especially of membrane phospholipids. Alterations to the length and saturation of membrane phospholipid fatty acids affect membrane fluidity and membrane receptor signaling [18–20]. Concurrent with impaired lipid metabolism, fructose overconsumption leads to oxidative stress and inflammation, both of which are also implicated in the pathogenesis of metabolic syndrome and its various complications [21,22].

One potential positive effect of physical activity on lipid metabolism could be the activation of brown adipose tissue (BAT) [23,24]. Because BAT is able to degrade high amounts of fatty acids and produce heat, it could be a good therapeutic target against obesity-associated disorders [25–28]. The relatively recent use of computerized tomography and positron emission tomography to confirm the presence of BAT in adult humans [29–31] has led to renewed scientific interest in this tissue.

Wistar rats have been bred for use as non-obese hereditary hypertriacylglycerolemic (HHTg) animals in a genetic model of hypertriacylglycerolemia. HHTg rats exhibit many of the symptoms associated with metabolic syndrome in humans: hypertriacylglycerolemia; increased liver and muscle TAG; muscle and adipose tissue resistance to insulin; hyperinsulinemia; impaired glucose tolerance; mildly elevated blood pressure; hyperuricemia; higher serum C-reactive protein [32]. A review of these symptoms can be found in the literature [33,34]. In HHTg rats, such symptoms are easy to exacerbate by high-sucrose feeding [35].

The purpose of this study was to test the effect of voluntary running (VR) wheel exercise on lipid metabolism disorders in non-obese HHTg rats fed with either a standard diet or, to intensify the symptoms of metabolic syndrome, a high-sucrose diet. To simulate sugar consumption in the human population, sucrose was administered in the form of a drink [36]. Our findings shed new light on the combined effects of physical activity and diet on genetically-induced dyslipidemia. We show that physical activity is able to reduce the negative effects of sucrose consumption. In particular, we focus on oxidative stress, lipid deposition in non-adipose tissue, the amount and composition of lipotoxic intermediates, the composition of membrane phospholipids, and BAT activity.

## Materials and Methods

### Animals

Male HHTg rats (3 months old) with free access to food and drink were housed in a temperature-controlled room (22°C) on a 12:12 h light-dark cycle. The rats were divided into 4 groups: the first group was fed a control diet without the possibility of voluntary running (CD, n = 8); the second group was fed a control diet with the possibility of voluntary running (CD+VR, n = 23); the third group was fed a high-sucrose diet without the possibility of voluntary running (HSD, n = 8); and the fourth group was fed a high-sucrose diet with the possibility of voluntary running (HSD+VR, n = 26). At the beginning of the experiment, all groups had the same average body weight (345 ± 10 g) and serum TAG concentration (7.6 ± 0.6 mM). All rats had continuous access to food and drink. The control diet consisted of 23% protein, 43% starch, 7% fat, 5% fibre and 1% vitamin and mineral mixture (standard chow diet, Bonagro, Czech Republic). For the HSD groups, the drink consisted of sucrose in drinking water (20% v/w sucrose solution). Rats without the possibility of running were placed in standard cages while rats in the VR groups had free access to a VR wheel at all times (Rat Activity Wheel and Cage, Panlab, Harvard Apparaturs, USA). The intensity of physical activity (i.e. number of wheel rotations) was recorded each day for every rat in the VR groups during the four weeks of the experiment. The characteristics of all rats used in the experiment are summarized in Table 1.

**Table 1:** Characteristics of rats used in experiment.

							ANOVA results		
	**CD**	**CD + VR**	P	**HSD**	**HSD + VR**	P	P VR	P HSD	P VR*HSD
Voluntary running (km/day)	-	Range: 1.65–11; Average: 6.12		-	Range: 2.48–8.24; Average: 5.5				
Food consumption (g/day)	24.6 ± 0.6	19.5 ± 0.4	0.01	12.3 ± 0.4	13.1 ± 0.3	NS	0.01	0.01	0,01
Drink consumption (ml/day)	34 ± 2.2	45.1 ± 2	0.01	59.6 ± 5	68.4 ± 6	NS	0.01	0.01	NS
Sucrose (% of energy)	-	-		53.2 ± 2.0	55.1 ± 4.9	NS			
Final body weight (g)	405 ± 6	386 ± 8	NS	417.8 ± 8	408 ± 7	NS	0.01	0.02	NS

Values for the consumption of food and drink were determined on days 7, 14 and 21 and did not differ significantly. Table results are based on all values. ‘Sucrose (% of energy)’ indicates the percentage of total energy (J) obtained from the sucrose. Abbreviations: ANOVA, two-way analysis of variance; CD, control diet; HSD, high-sucrose diet; VR, voluntary running.

In order to create homogenous CD+VR and HSD+VR groups, only animals with a comparable intensity of physical activity (5–8 km per day) were selected for metabolic analyses. However, the correlation analyses are based on the entire cohort of animals. At the end of the study, all rats were moved to standard cages for one night; here they did not have the possibility of running, but still had free access to food and drink. The following day they were decapitated, and their serum and tissues collected for final biochemical analyses. All experiments were performed in accordance with the Animal Protection Law of the Czech Republic 311/1997, which is in compliance with European Community Council recommendations (86/609/ECC) for the use of laboratory animals, and approved by the ethical committee of the Institute for Clinical and Experimental Medicine, Prague.

### Biochemical parameters

Blood glucose levels were measured by glucose oxidase assay (GLU GOD, Erba-Lachema, Czech Republic) using tail vein blood drawn into 5% trichloracetic acid and promptly centrifuged. Serum TAG concentrations were quantified using standard enzymatic methods (TG L 250 S, Erba-Lachema, Czech Republic). Non-esterified fatty acid levels (NEFA) were determined using an acyl-CoA oxidase-based colorimetric kit (Roche Diagnostics GmbH, Germany). Serum insulin concentrations were evaluated using a rat insulin enzyme-linked immunosorbent assay kit (Mercodia, Sweden). Irisin serum concentrations were measured using an irisin enzyme-linked immunosorbent assay kit (Irisin ELISA, BioVendor, Czech Republic). The insulin sensitivity index (ISI) was calculated from fasting insulin (FI) and fasting glucose (FG) as described previously [37]. ISI = 1/(FG*FI), where FG is expressed as mg/dl and FI as mIU/l.

### Adipose tissue protein content

The protein content of both white and brown adipose tissue was determined according to the Folin–Ciocalteu method [38]. Prior to analysis, the samples were disintegrated by boiling in 1 M NaOH and then filtered.

### Lipolysis in isolated epididymal adipose tissue

The measurement of basal and epinephrine-stimulated lipolysis was performed as described previously [39]. The distal parts of the epididymal adipose tissue were incubated in Krebs-Ringer phosphate buffer containing 3% bovine serum albumin (BSA) fraction V (Sigma, USA) at 37°C, pH 7.4, with or without epinephrine (1.37 μM). The tissue was incubated for 2 h and the NEFA concentrations were measured.

### Oxidative stress parameters

The activities of superoxide dismutase (SOD) and glutathione peroxidase (GSH-Px) were determined by the commercially available kits, Superoxide Dismutase Assay Kit and Glutathione Peroxidase Assay Kit (Cayman Chemical Company, USA). The concentration of thiobarbituric acid reactive substances (TBARS) was quantified as described previously [40] spectrophotometrically by the reaction with thiobarbituric acid. All parameters were adjusted to the tissue protein concentration and plasma volume.

### Tissue triacylglycerols measurements

Tissues were disintegrated using a tissue homogenizer and TAG extracted for 16 h in trichloromethane:methanol (2:1), after which 2% KH_2_PO_4_ was added. After 24 h, the mixture was centrifuged, and the organic phase removed and evaporated. The resulting pellet was dissolved in 100 μl of isopropyl alcohol and the TAG content determined by enzymatic assay (TG L 250 S, Erba-Lachema, Czech Republic). From this TAG solution, 10 μl was pipetted into 1 ml of enzymatic assay solution and intensively mixed for 10 s [22].

### Magnetic resonance spectroscopy

The intramyocelullar and extramyocellular lipid (IMCL and EMCL) content of the skeletal muscle was determined by ^1^H magnetic resonance spectroscopy (MRS) as previously described [41,42]. The analysis of muscle lipids was performed using a PRESS sequence with the following measurement parameters: repetitive time TR = 2500 ms; echo time TE = 20 and 135 ms. During the measurement, the animals underwent anesthesia with isoflurane. The determination was carried out on an experimental Bruker Biospec 47/20 tomograph (Bruker, Germany) with a magnetic induction of 4.7 T. A volume of interest of 5 x 2 x 13 mm was placed into the gastrocnemius muscle. The measured spectra were further processed using LC Model computer software. The detected quantities of the IMCL-CH_2_ and EMCL-CH_2_ groups were related to the signal of the N-methyl group of creatine/phosphocreatine, which was assessed from the same NMR spectra. Then, the percentage content of IMCL in the total lipids was calculated.

### Content and composition of ceramides and diacylglycerols

Ceramides and diacylglycerols (DAG) were measured according to the methods as previously described [43,44]. Briefly, lipids were extracted from ~20mg of tissue using an extraction mixture consisting of isopropanol:water:ethyl acetate (35:5:60; v:v:v). The ceramides and DAG were measured using an Agilent 6460 triple quadrupole mass spectrometer. Both sphingolipids and DAG were analyzed using a positive ion electrospray ionization source with multiple reaction monitoring. Chromatographic separation was performed using an Agilent 1290 Infinity Ultra Performance Liquid Chromatograph. The analytical column was a reverse-phase Zorbax SB-C8, 2.1x150 mm, 1.8 μm (Agilent, USA). The separation was conducted in a binary gradient using 2 mM ammonium formate, 0.15% formic acid in methanol as Solvent A and 1.5 mM ammonium formate, 0.1% formic acid in water as Solvent B at a flow rate of 0.4 ml/min. C17:0-ceramide and 1,3-dipentadecanoyl-rac-glycerol (Avanti Polar Lipids, USA) were used as the internal standards.

### Fatty acid composition of membrane phospholipids

The extraction, separation and methylation of skeletal muscle lipids were performed as previously described [45]. Muscle total lipids were extracted by trichlormethane:methanol (2:1) using a modified Folch method [46]. Phospholipids were isolated by thin layer chromatography using hexane-diethylether-acetic acid (80:20:3 v/v) as the solvent system. The fatty acid in the muscle phospholipids was converted to methyl esters using a solution of 1% Na in methanol. The fatty acid methyl esters were eluted with hexane and separated by gas chromatography (HP 5890A GC, USA) using hydrogen as the carrier gas. The proportions of fatty acids were relative to the sum of fatty acids analyzed. The indexes of desaturase activity were assessed by calculating the product/precursor ratios as follows: 20:4n-6/20:3n-6 for Δ-5 desaturase; 18:3n-6/18:2n-6 for Δ-6 desaturase.

### Brown adipose tissue activity

BAT activity was determined according to the rate of palmitate oxidation to CO_2_ *ex vivo*. After decapitation, the interscapular BAT was divided into parts, one of which was immediately incubated for 2 h in 5 ml of Krebs-Ringer bicarbonate buffer, pH 7.4. The buffer contained 1 mM unlabeled palmitate, 18.5 kBq/ml of ^14^C-palmitate (Perkin Elmer, USA) and 3 mg/ml BSA fraction V (Sigma, USA). The incubation was performed in a 95% O_2_ + 5% CO_2_ atmosphere in sealed vials, the central area of which was separated from the medium. After 2 h incubation, the tissue was removed and 0.2 ml of 1 M hyamine hydroxide was injected into the central compartment of the incubation vessel, with 0.5 ml of 0.5 M H_2_SO_4_ added to the incubation medium in order to free the CO_2_. The sealed vessels were incubated for another 45 min, this time in an air atmosphere. The hyamine hydroxide with absorbed CO_2_ was then quantitatively transferred into a scintillation vial containing 10 ml of toluene-based scintillation fluid for radioactivity counting [47].

### Statistical analysis

Statistical analyses were performed using Statistica^TM^ software (StatSoft) with the data expressed as mean ± SEM. Two-way analysis of variance (ANOVA) was applied to test the effects of VR, HSD and the interaction of these two factors. Because ANOVA was applied to all groups, if a difference in the effect of VR was observed in only one dietary group, the result would appear to be insignificant. To avoid such an oversight, we also used the Student *t* test to compare CD against CD+VR and HSD against HSD+VR. The correlations were tested using Pearson's coefficient, with statistical significance being defined as *P* < 0.05.

## Results

### Voluntary running had a positive effect on white adipose tissue and serum parameters

To analyze the effect of voluntary running (VR) on white adipose tissue and serum, we selected 7 rats with a comparable intensity of physical activity (5 to 8 km/day) from both dietary groups. The results are summarized in Table 2. The white adipose tissue of the CD+VR rats had significantly better properties than that of the CD rats: reduced perirenal and epididymal adipose tissue; increased protein content; increased basal and adrenaline-stimulated lipolysis in the epididymal fat depot. The serum parameters of the CD+VR group were also dramatically better than those of the CD group: reduced fasting glycemia; a 60% decrease in postprandial and fasting TAG; a 45% reduction in postprandial NEFA; a 45% decrease in insulin concentration.

**Table 2:** Effect of voluntary running on metabolic characteristics of experimental groups.

							ANOVA results		
Parameter	**CD**	**CD + VR**	P	**HSD**	**HSD + VR**	P	P VR	P HSD	P VR*HSD
TAG fasted (mM)	5.54 ± 0.45	2.18 ± 0.36	0.01	8.57 ± 0.6	4.41 ± 0.46	0.01	0.01	0.01	NS
TAG non-fasting (mM)	8.0 ± 0.43	3.61 ± 0.28	0.01	14.33 ± 0.96	8.98 ± 0.55	0.01	0.01	0.01	NS
NEFA non-fasting (mM)	1.46 ± 0.156	0.8 ± 0.07	0.01	1.56 ± 0.2	1.01 ± 0.08	0.02	0.01	NS	NS
Glycemia fasted (mM)	5.27 ± 0.19	4.46 ± 0.28	0.03	5.16 ± 0.31	5.11 ± 0.51	NS	NS	NS	NS
Glycemia non-fasting (mM)	8.54 ± 0.39	8.45 ± 0.6	NS	8.6 ± 0.48	7.7 ± 0.44	NS	NS	NS	NS
Insulin non-fasting (nM)	0.537 ± 0.045	0.291 ± 0.017	0.03	0.757 ± 0.12	0.393 ± 0.059	0.05	0.01	0.03	NS
ISI	0.423 ± 0.017	0.638 ± 0.047	0.01	0.272 ± 0,021	0.421 ± 0.033	0.01	0.01	0.01	NS
Irisin non-fasting (nM)	0.137 ± 0.001	0.139 ± 0.039	NS	0.075 ± 0,044	0,087 ± 0.026	NS	NS	NS	NS
PRAT weight (g/100g)	2.29 ± 0.07	0.938 ± 0.104	0.01	2.57 ± 0.1	1.39 ± 0.12	0.01	0.01	0.01	NS
EAT weight (g/100g)	1.75 ± 0.06	0.895 ± 0.046	0.01	1.87 ± 0.09	1.17 ± 0.08	0.01	0.01	0.05	NS
EAT Protein content (%)	0.977 ± 0.044	1.478 ± 0.05	0.01	0.946 ± 0.049	1.2 ± 0.068	0.02	0.01	NS	0.03
EAT lipolysis basal (μmol(NEFA)/g/2h)	2.29 ± 0.28	4.52 ± 0.38	0.01	1.91 ± 0.37	2.98 ± 0.48	NS	0.01	0.05	NS
EAT lipolysis adrenaline (μmol(NEFA)/g/2h)	2.7 ± 0.14	6.21 ± 0.44	0.01	3.4 ± 0.21	6.26 ± 0.68	0.01	0.01	NS	NS

The data are expressed as mean ± SEM; n = 7, running intensity = 5–8 km/day. Abbreviations: ANOVA, two-way analysis of variance; CD, control diet; EAT, epididymal adipose tissue; HSD, high-sucrose diet; ISI, insulin sensitivity index; NEFA, non-esterified fatty acids; PRAT, perirenal adipose tissue; TAG, triacylglycerols; VR, voluntary running.

Although, as expected, these parameters were negatively affected by sucrose feeding, the same trends were observed for the white adipose tissue of the HSD+VR rats compared with that of the HSD ones. Indeed, the serum parameters of the HSD+VR rats were also significantly better than those of their control group: a 40% and 50% decrease in postprandial TAG and fasting TAG, respectively; a 35% reduction in postprandial NEFA; a 45% decrease in insulin concentration. In both dietary groups, VR did not influence either postprandial glucose blood concentration or serum irisin content.

To analyze the effect of VR and sucrose feeding on the insulin resistance of the rats, the insulin sensitivity index (ISI) was determined. Although the ISI was lower in the HSD-fed rats, it was still approximately 50% higher in both VR groups compared with their controls.

### Voluntary running had a positive effect on the markers of oxidative stress

Because both sucrose and physical activity increase the production of reactive oxygen species, we measured the effect of VR on various markers of oxidative stress. The activity of two antioxidant enzymes, superoxide dismutase (SOD) and glutathione peroxidase (GSH-PX), was determined. And, to monitor lipid peroxidation, thiobarbituric acid reactive substances (TBARS) were quantified. All three markers were measured in the liver, heart, soleus muscle and plasma. Overall, the CD-fed rats were less sensitive to oxidative stress, but VR was beneficial to both groups (Table 3).

**Table 3:** Effect of voluntary running on oxidative stress parameters.

							ANOVA results		
	**CD**	**CD + VR**	P	**HSD**	**HSD + VR**	P	P VR	P HSD	P VR*HSD
**Liver**									
SOD (U/mg_prot_)	0.162 ± 0.017	0.193 ± 0.015	NS	0.094 ± 0.006	0.148 ± 0.014	0.01	0.01	0.01	NS
GSH-PX (µmol_NADPH_/min/g_prot_)	281 ± 16	292 ± 22	NS	239 ± 15	234 ± 20	NS	NS	0.02	NS
TBARS (nmol/mg_prot_)	1.129 ± 0.097	1.231 ± 0.129	NS	1.788 ± 0.116	1.753 ± 0.269	NS	NS	0.01	NS
**Heart**									
SOD (U/mg_prot_)	0.033 ± 0.005	0.038 ± 0.002	NS	0.041 ± 0.004	0.038 ± 0.003	NS	NS	NS	NS
GSH-PX (µmol_NADPH_/min/g_prot_)	93 ± 4	108 ± 5	0.05	70 ± 5	85 ± 3	0.04	0.01	0.01	NS
TBARS (nmol/mg_prot_)	0.399 ± 0.017	0.319 ± 0.018	0.02	0.479 ± 0.014	0.514 ± 0.017	NS	NS	0.01	0.01
**Soleus**									
SOD (U/mg_prot_)	0.145 ± 0.013	0.112 ± 0.014	NS	0.131 ± 0,03	0.135 ± 0.013	NS	NS	NS	NS
GSH-PX (µmol_NADPH_/min/g_prot_)	53 ± 6	75 ± 6	0.02	58 ± 5	76 ± 7	NS	0.01	NS	NS
TBARS (nmol/mg)	2.081 ± 0.16	1.622 ± 0.082	0.04	2.246 ± 0.23	1.604 ± 0.191	0.05	0.01	NS	NS
**Plasma**									
SOD (U/ml)	2.676 ± 0.09	2.691 ± 0.157	NS	1.182 ± 0.16	2.277 ± 0.116	0.01	0.01	0.01	0.01
GSH-PX (µmol_NADPH_/min/l)	296 ± 12	280 ± 14	NS	225 ± 19	282 ± 10	0.04	NS	0.03	0.02
TBARS (nmol/ml)	1.862 ± 0.081	1.446 ± 0.114	0.02	2.177 ± 0.132	2.0 ± 0.056	NS	0.01	0.01	NS

The data are expressed as mean ± SEM; n = 6, running intensity = 5–8 km/day. U: One unit of SOD is defined as the amount of the enzyme needed to exhibit 50% dismutation of the superoxide radical. Abbreviations: ANOVA, two-way analysis of variance; CD, control diet; GSH-PX, glutathione peroxidase; HSD, high-sucrose diet; SOD, superoxide dismutase; TBARS, thiobarbituric acid reactive substances; VR, voluntary running.

### Voluntary running reduced the content of tissue triacylglycerols (TAG)

The amount of triacylglycerols (TAG) stored in the liver, muscles, aorta and kidney were determined (Table 4). Only in the liver of the HSD rats was found a significantly higher amount of tissue TAG. Compared with their controls, the CD+VR and HSD+VR groups exhibited considerably reduced TAG in all of the studied tissues. ^1^H MRS was used to study how VR affected the ratio of intramyocellular lipids (IMCL) to total muscle lipid content in the skeletal gastrocnemius muscle (Table 4). The IMCL proportion was reduced by 35% in the CD+VR group compared its CD control. In contrast, this effect was not found in the HSD-fed rats.

**Table 4:** Effect of voluntary running on triacylglycerol accumulation in tissues.

							ANOVA results		
Tissue	**CD**	**CD + VR**	P	**HSD**	**HSD + VR**	P	P VR	P HSD	P VR*HSD
Liver TAG (µmol/g)	14.63 ± 1.37	7.74 ± 0.44	0.01	19.23 ± 1.63	12.24 ± 0.57	0.01	0.01	0.01	NS
*m*. *gastrocnemius* TAG (µmol/g)	5.82 ± 0.8	1.67 ± 0.23	0.01	5.54 ± 0.61	2.73 ± 0.19	0.01	0.01	NS	NS
*m*. *gastrocnemius* IMCL (%)	43 ± 6	28 ± 11	NS	48 ± 9	48 ± 14	NS	NS	NS	NS
Heart TAG (µmol/g)	2.2 ± 0.25	1.53 ± 0.23	0.05	2.75 ± 0.3	1.9 ± 0.17	0.05	0.05	NS	NS
Diaphragm TAG (µmol/g)	35.1 ± 2.7	24.3 ± 0.5	0.03	39.4 ± 4.2	27.9 ± 2.8	0.05	0.01	NS	NS
Aorta TAG (µmol/g)	58.5 ± 4.7	35.5 ± 8.1	0.05	89.7 ± 7.5	33.4 ± 4.6	0.01	0.01	NS	0.04
Kidney TAG (µmol/g)	6.17 ± 0.54	4.14 ± 0.43	0.02	7.54 ± 1.1	3.66 ± 0.51	0.01	0.01	NS	NS

The data are expressed as mean ± SEM; n = 7, running intensity = 5–8 km/day. Abbreviations: ANOVA, Two-way analysis of variance; CD, control diet; HSD, high-sucrose diet; IMCL, intramyocellular lipids; TAG, triacylglycerols; VR, voluntary running.

### Voluntary running reduced the content and altered the composition of tissue diacylglycerols, but had no effect on ceramide content or composition

The results reported above indicate that VR significantly lowered TAG storage in the liver, skeletal muscles, heart, kidney and aorta. Because tissue TAGs are precursors of lipotoxic intermediates, primarily ceramides and diacylglycerols (DAG), we analyzed the effect of VR on the concentrations of these intermediates in the liver, heart and soleus muscle. High-sucrose feeding increased the total DAG content in the tissues (Table 5). Interestingly, while VR was beneficial to the liver and heart of both groups, it was only beneficial to the soleus muscle of the HSD+VR rats (Table 5).

**Table 5:** Effect of voluntary running on diacylglycerol content in tissues.

							ANOVA results		
DAG (pmol/mg tissue)	**CD**	**CD + VR**	P	**HSD**	**HSD + VR**	P	P VR	P HSD	P VR*HSD
**Liver**									
18:2/18:2	115.6 ± 19	189.9 ± 31.1	NS	95.8 ± 6.7	92 ± 3.2	NS	NS	0.05	NS
16/18:2	126.5 ± 37	102.1 ± 11.6	NS	73.1 ± 5.6	81.4 ± 42.1	NS	NS	NS	NS
18:1/18:2	40.7 ± 2.2	37.4 ± 7	NS	42.7 ± 1.7	36.0 ± 1	0.05	NS	NS	NS
16/16	99.2 ± 6.7	52.4 ± 5.8	0.01	144.6 ± 10.4	55.3 ± 2.3	0.02	0.01	0.02	0.03
16/18:1	873.1 ± 61	572.4 ± 51.8	0.02	1747.5 ± 35.3	783.1 ± 71.4	0.02	0.01	0.01	0.01
18:1/18:1	114.1 ± 15.8	46.9 ± 9.5	0.03	195.4 ± 32.5	141.4 ± 22.9	NS	0.02	0.01	NS
18/18:1	45.4 ± 6.8	23.0 ± 1.9	0.05	77.2 ± 9.9	32 ± 5.7	0.04	0.01	0.03	NS
Total	1414.7 ± 84.5	1024.1 ± 82.5	0.03	2376.2 ± 70.8	1221.3 ± 136	0.04	0.01	0.01	0.01
**Heart**									
18:2/18:2	66 ± 4.7	66.6 ± 4.6	NS	38 ± 5.3c	57.6 ± 7.8	NS	NS	0.02	NS
16/18:2	49.4 ± 10.4	31.2 ± 1.3	NS	29.2 ± 6.5	43.8 ± 10.1	NS	NS	NS	NS
18:1/18:2	41.3 ± 0.8	34.7 ± 2.1	0.05	30.5 ± 1.4	38.4 ± 4.9	NS	NS	NS	0.05
16/16	105.4 ± 13.7	77.4 ± 11.8	NS	131.3 ± 11.4	69.1 ± 5.4	0.02	0.01	NS	NS
16/18:1	339.9 ± 24.5	210.6 ± 10	0.03	440 ± 22.6	329.5 ± 18.3	0.02	0.01	0.01	NS
18:1/18:1	44.9 ± 2.4	24.8 ± 3.5	0.01	89.2 ± 10.2	78.05 ± 12.3	NS	NS	0.01	NS
18/18:1	46.4 ± 9	37.3 ± 4.5	NS	78.6 ± 7.8	42.6 ± 2.1	0.04	0.01	0.01	0.05
Total	693.3 ± 37.3	482.6 ± 23.2	0.02	836.8 ± 33.7	659.1 ± 43.8	0.03	0.01	0.01	NS
**Soleus**									
18:2/18:2	89.6 ± 15.2	114.8 ± 8.9	NS	58.5 ± 4.0	78.6 ± 14.1	NS	NS	0.05	NS
16/18:2	43.9 ± 8.5	42.7 ± 6.6	NS	40.3 ± 14.7	40.8 ± 14.7	NS	NS	NS	NS
18:1/18:2	28.1 ± 5.3	30.7 ± 2.6	NS	30.6 ± 7.5	28.8 ± 7.0	NS	NS	NS	NS
16/16	80.85 ± 8.7	62.95 ± 1.8	0.02	103.5 ± 4.0	85.8 ± 14.8	NS	0.02	0.01	NS
16/18:1	208.95 ± 21.2	178.1 ± 5.0	NS	321.4 ± 25.3	259.2 ± 13.9	0.05	0.04	0.01	NS
18:1/18:1	67.8 ± 11.3	58.5 ± 3.4	NS	132.2 ± 4.8	87.4 ± 8.7	0.05	0.02	0.01	NS
18/18:1	17.85 ± 2.3	17.6 ± 1.7	NS	29.97 ± 0.5	21.3 ± 2.5	0.05	NS	0.01	NS
Total	537.1 ± 57.1	505.3 ± 26.3	NS	716.55 ± 8.2	601.8 ± 55.6	0.05	NS	0.04	NS

The data are expressed as mean ± SEM; n = 4, running intensity = 5–8 km/day. Abbreviations: ANOVA, Two-way analysis of variance; CD, control diet; DAG, diacylglycerol; HSD, high-sucrose diet; VR, voluntary running.

**Fig 1:**
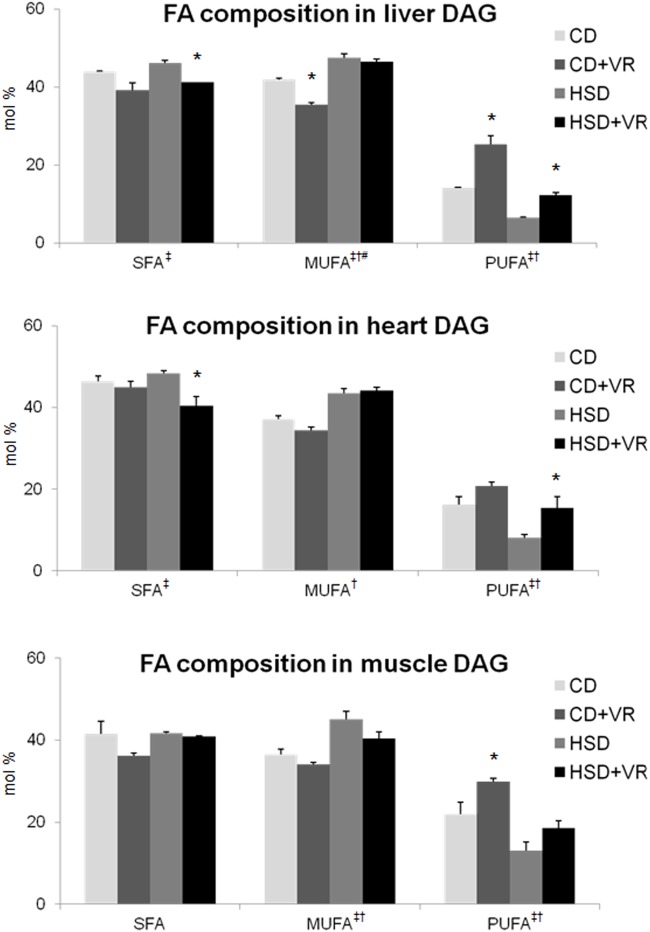
Effect of voluntary running on the proportion of fatty acids in tissue DAG. Individual species of DAG were analyzed in the liver, heart and soleus muscle of rats from all groups; in the case of the VR groups the rats were selected on the basis of the intensity of their activity (5–8 km/day). From these results, the percentage proportions of saturated fatty acids (SFA expressed as 18:0 + 16:0), monounsaturated fatty acids (MUFA, 18:1) and polyunsaturated fatty acids (PUFA, 18:2) in tissue DAG were calculated. The data are expressed as mean ± SEM, * significant effect of voluntary running within the dietary groups (CD+VR against CD; HSD+VR against HSD); Two-way ANOVA results: ‡ significant effect of VR, † significant effect of HSD, # significant interaction between VR and HSD; *P* < 0.05. Abbreviations: DAG, diacylglycerol; HHTg, hereditary hypertriacylglycerolemic rats; HSD, high-sucrose diet; VR, voluntary running.

Based on the tissue content of individual DAG subspecies, we calculated the proportion of saturated fatty acids (16:0 + 18:0), monounsaturated fatty acids (18:1) and polyunsaturated fatty acids (18:2) in tissue DAG (Fig 1). VR led to a strong increase in polyunsaturated fatty acids in both dietary groups. This increase was accompanied by a small decrease in the proportion of saturated and monounsaturated fatty acids.

The effect of VR on the ceramide content in the tissues was less pronounced compared with its effect on tissue DAG. Although HSD led to an increased amount of several ceramides in the liver, heart and soleus muscle, VR had almost no effect on the tissue ceramide content of either group (Table 6).

**Table 6:** Effect of voluntary running on ceramide content in tissues.

							ANOVA results		
Ceramide (pmol/mg tissue)	**CD**	**CD + VR**	P	**HSD**	**HSD + VR**	P	P VR	P HSD	P VR*HSD
**Liver**									
C14-Cer	0.301 ± 0.058	0.221 ± 0.35	NS	0.231 ± 0.049	0.242 ± 0.05	NS	NS	NS	NS
C16-Cer	6.802 ± 0.904	7.233 ± 1.818	NS	4.031 ± 0.420	5.222 ± 0.811	NS	NS	NS	NS
C18:1-Cer	0.022 ± 0.003	0.018 ± 0.003	NS	0.034 ± 0.002	0.024 ± 0.004	0.05	0.05	0.01	NS
C18-Cer	0.449 ± 0.088	0.351 ± 0.082	NS	0.457 ± 0.07	0.4 ± 0.025	NS	NS	NS	NS
C20-Cer	0.083 ± 0.008	0.053 ± 0.01	0.05	0.125 ± 0.02	0.067 ± 0.01	0.05	0.01	0.05	NS
C22-Cer	0.868 ± 0.07	0.813 ± 0.265	NS	1.099 ± 0.065	0.786 ± 0.227	NS	NS	NS	NS
C24:1-Cer	3.013 ± 0.327	2.571 ± 0.376	NS	3.184 ± 0.103	3.242 ± 0.632	NS	NS	NS	NS
C24-Cer	6.284 ± 0.235	10.7 ± 3.8	NS	11.29 ± 1.19	7.72±2.13	NS	NS	NS	NS
Total	17.82 ± 1.29	21.928 ± 3.76	NS	20.46 ± 1.7	17.7 ± 3	NS	NS	NS	NS
**Heart**									
C14-Cer	0.189 ± 0.03	0.109 ± 0.006	0.05	0.245 ± 0.023	0.187 ± 0.028	NS	0.02	0.02	NS
C16-Cer	4.81 ± 1.02	5.1 ± 0.126	NS	5.63 ± 0.77	5.924 ± 0.926	NS	NS	NS	NS
C18:1-Cer	0.018 ± 0.002	0.018 ± 0.002	NS	0.033 ± 0.001	0.029 ± 0.004	NS	NS	0.01	NS
C18-Cer	2.110 ± 0.134	1.962 ± 0.263	NS	1.937 ± 0.376	2.058 ± 0.388	NS	NS	NS	NS
C20-Cer	1.334 ± 0.045	1.421 ± 0.050	NS	1.738 ± 0.296	1.648 ± 0.147	NS	NS	NS	NS
C22-Cer	1.609 ± 0.147	1.878 ± 0.052	NS	1.995 ± 0.284	1.665 ± 0.136	NS	NS	NS	NS
C24:1-Cer	1.119 ± 0.079	1.08 ± 0.064	NS	1.995 ± 0.218	1.636 ± 0.148	NS	NS	0.01	NS
C24-Cer	8.92 ± 0.6	10.06 ± 1.011	NS	10.9 ± 1.2	8.67 ± 0.718	NS	NS	NS	NS
Total	20.1 ± 0.78	21.64 ± 1.04	NS	24.49 ± 1.68	21.82 ± 1.6	NS	NS	NS	NS
**Soleus**									
C14-Cer	0.193 ± 0.012	0.137 ± 0.01	0.02	0.230 ± 0.01	0.258 ± 0.17	NS	NS	0.01	0.01
C16-Cer	4.672 ± 0.147	5.847 ± 0.691	NS	6.539 ± 0.185	6.530 ± 1.131	NS	NS	0.04	NS
C18:1-Cer	0.041 ± 0.002	0.041 ± 0.002	NS	0.049 ± 0.006	0.051 ± 0.003	NS	NS	0.02	NS
C18-Cer	3.387 ± 0.523	4.119 ± 0.598	NS	3.394 ± 0.968	4.649 ± 1.693	NS	NS	NS	NS
C20-Cer	0.535 ± 0.07	0.684 ± 0.009	NS	0.654 ± 0.093	0.827 ± 0.098	NS	0.05	NS	NS
C22-Cer	0.344 ± 0.021	0.425 ± 0.031	NS	0.389 ± 0.034	0.439 ± 0.044	NS	NS	NS	NS
C24:1-Cer	0.610 ± 0.032	0.707 ± 0.051	NS	0.765 ± 0.077	0.910 ± 0.065	NS	NS	0.01	NS
C24-Cer	2.61 ± 0.19	2.71 ± 0.22	NS	3.34 ± 0.46	4.13 ± 0.024	NS	NS	0.01	NS
Total	12.39 ± 0.83	14.63 ± 0.82	NS	15.36 ± 1.3	17.79 ± 2.74	NS	NS	NS	NS

The data are expressed as mean ± SEM; n = 4, running intensity = 5–8 km/day. Abbreviations: ANOVA, Two-way analysis of variance; CD, control diet; HSD, high-sucrose diet; VR, voluntary running.

### Voluntary running positively modulated the composition of fatty acids in muscle membrane phospholipids

We studied the effect of VR on the fatty acid composition of membrane phospholipids in the soleus muscle. Several important results were obtained. Palmitooleic acid (16:1n7) increased after HSD feeding, but decreased after VR (Table 7). Also after VR, the Δ-5 desaturase activity index increased in both dietary groups. Furthermore, VR had a positive effect on the proportions of n-6 fatty acids.

**Table 7:** Effect of voluntary running on fatty acid composition of soleus muscle membrane phospholipids.

							ANOVA results		
Fatty acid (mol %)	**CD**	**CD + VR**	P	**HSD**	**HSD + VR**	P	P VR	P HSD	P VR*HSD
14:00	0.155 ± 0.050	0.143 ± 0.055	NS	0.132 ± 0.050	0.074 ± 0.013	NS	NS	NS	NS
16:00	15.03 ± 1.66	14.61 ± 1.17	NS	15.58 ± 3.65	12.66 ± 0.88	NS	NS	NS	NS
18:00	18.17 ± 1.59	20.02 ± 0.7	NS	19.17 ± 0.76	19.9 ± 0.3	NS	NS	NS	NS
Σ SFA	33.37 ± 2.5	34.8 ± 0.73	NS	34.92 ± 2,97	32.66 ± 0.9	NS	NS	NS	NS
16: 1n7	1.255 ± 0.219	0.666 ± 0.096	0.05	1.876 ± 0.587	1.135 ± 0.09	0.05	0.02	0.05	NS
18: 1n7	3.628 ± 0.705	3.998 ± 0.198	NS	4.094 ± 0,422	4.331 ± 0,260	NS	NS	NS	NS
18: 1n9	4.517 ± 0.506	5.079 ± 0.155	NS	6.53 ± 0.741	6.188 ± 0.273	NS	NS	0.01	NS
Σ MUFA	9.42 ± 1.54	9.84 ± 0.41	NS	12,54 ± 1.2	11.69 ± 0,27	NS	NS	0.02	NS
18: 2n-6	20.53 ± 1.15	22.72 ± 0.47	0.05	22.78 ± 0.6	21.29 ± 0.741	NS	NS	NS	0.01
18: 3n-6	0.151 ± 0. 009	0.172 ± 0.004	0.05	0.140 ± 0.005	0.125 ± 0.004	0.05	NS	0.01	0.01
20: 2n-6	0.211 ± 0.020	0.262 ± 0.013	NS	0.260 ± 0.041	0.276 ± 0,024	NS	NS	NS	NS
20: 3n-6	0.984 ± 0.176	0.678 ± 0.023	0.02	0.879 ± 0.103	0.832 ± 0.080	NS	0.02	NS	0.05
20: 4n-6	20.48 ± 2.66	18.02 ± 0.73	NS	16.9 ± 1.77	18.78 ± 0.67	NS	NS	NS	0.05
Σ n-6 PUFA	43.08 ± 1.89	42.4 ± 0.98	NS	41.48 ± 2.45	42.02 ± 0.5	NS	NS	NS	NS
18: 3n-3	0.136 ± 0.095	0.334 ± 0.113	NS	0.199 ± 0.154	0.269 ± 0.105	NS	NS	NS	NS
20: 5n-3	0.254 ± 0.068	0.193 ± 0.018	NS	0.242 ± 0.035	0.179 ± 0.018	NS	0.04	NS	NS
22: 5n-3	5.255 ± 0.763	4.281 ± 0.205	NS	4.021 ± 0.659	4.613 ± 0.312	NS	NS	NS	0.03
22: 6n-3	8.488 ± 1.294	7.624 ± 0.393	NS	6.593 ± 0.995	8.576 ± 0.479	NS	NS	NS	0.03
Σ n-3 PUFA	14.13 ± 2.06	12.6 ± 0.52	NS	11.06 ± 1.63	13.64 ± 0.85	NS	NS	NS	NS
Δ-5 desaturase index	21.16 ± 1.050	26.79 ± 1.35	0.05	19.3 ± 0.45	23.4 ± 1.83	0.05	0.02	NS	NS
Δ-6 desaturase index	0.0074 ± 0.001	0.0076 ± 0.001	NS	0.0062 ± 0.0001	0.0059 ± 0.0002	NS	NS	0.01	NS

The data are expressed as mean ± SEM; n = 5, running intensity: 5–8 km/day. Abbreviations: ANOVA, Two-way analysis of variance; CD, control diet; HSD, high-sucrose diet; MUFA, monounsaturated fatty acids; PUFA, polyunsaturated fatty acids; SFA, saturated fatty acids; VR, voluntary running.

### Voluntary running affected the parameters of brown adipose tissue (BAT)

We studied the effect of VR on interscapular BAT metabolism (Table 8). No significant differences were found between the interscapular BAT weights of the four groups. *Ex vivo*, compared with its control, CD+VR exhibited a significantly increased rate of exogenous albumin-bound palmitate oxidation to CO_2_; this effect was not significant in the HSD-fed rats. VR also led to increased protein content in the BAT of the CD+VR group compared with CD.

**Table 8:** Effect of voluntary running on the parameters of interscapular brown adipose tissue.

							ANOVA results		
Parameter	**CD**	**CD + VR**	P	**HSD**	**HSD + VR**	P	P VR	P HSD	P VR*HSD
weight (mg/100g)	0.54 ± 0.084	0.62 ± 0.081	NS	0.51 ± 0.047	0.58 ± 0.044	NS	NS	NS	NS
PA oxidation into CO_2_ (nmol/g/2h)	58.2 ± 9	83.4 ± 11.1	0.05	72.2 ± 12.5	85.3 ± 16.9	NS	0.04	NS	NS
Protein content (%)	5.52 ± 0.27	7.12 ± 0.46	0.01	5.17 ± 0.37	5.73 ± 0.4	NS	0.02	NS	NS

The data are expressed as mean ± SEM; n = 7, running intensity = 5–8 km/day. Abbreviations: ANOVA, Two-way analysis of variance; CD, control diet; HSD, high-sucrose diet; PA, palmitic acid; VR, voluntary running.

### Correlation analysis

**Fig 2:**
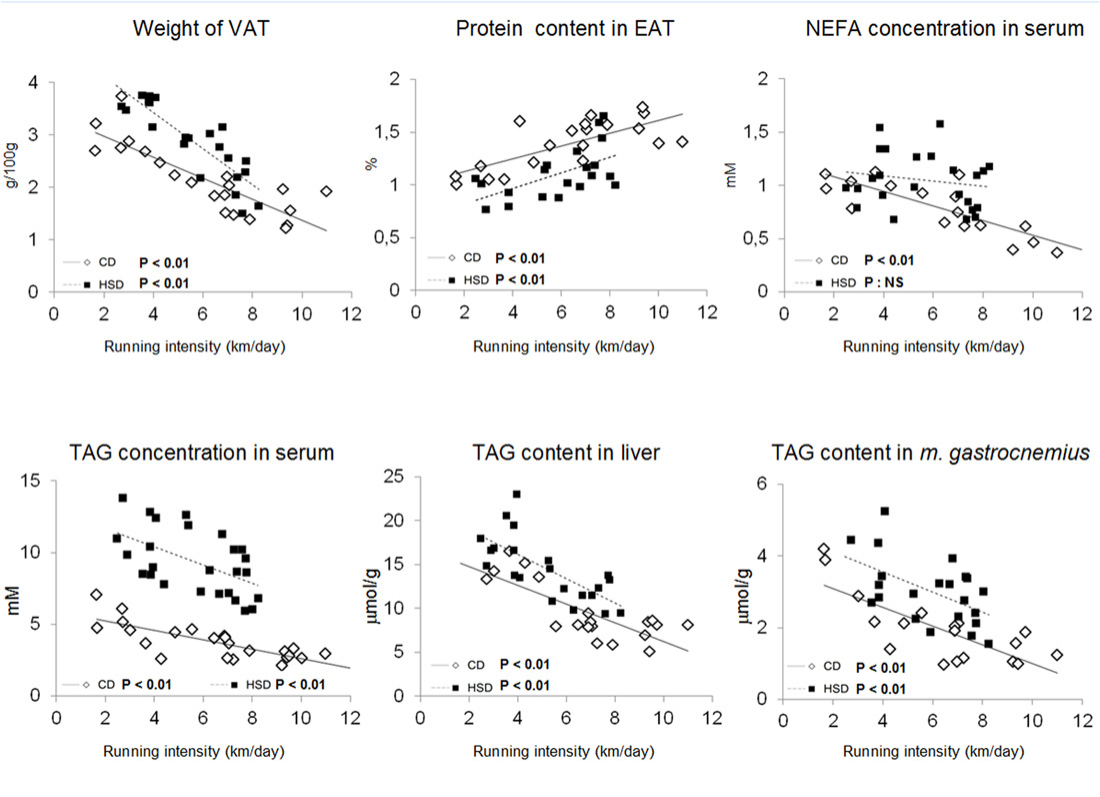
Correlation between intensity of physical activity and selected parameters of metabolic syndrome. The experimental rats had the possibility of voluntary running in a rat wheel. Physical activity was monitored every day. The plots show the dependence of selected parameters at experiment end on the average daily intensity of physical activity. Abbreviations: EAT, epididymal adipose tissue; NEFA, non-esterified fatty acids; PRAT, perirenal adipose tissue; TAG, triacylglycerols.

To explore the effect of VR on lipid metabolism, we analyzed the dependence of lipid metabolism parameters on the intensity of physical activity. For the CD+VR group, negative correlations were found between the intensity of physical activity and (i) the serum concentrations of TAG and NEFA (ii) the TAG content in the muscle and liver, and (iii) the combined weight of the epididymal and perirenal fat depots (Fig 2). Furthermore, we found a positive correlation between the intensity of physical activity and protein content in epididymal adipose tissue. Apart from the serum NEFA concentration, all of these correlations were also observed for the HSD+VR group.

## Discussion

To study the effect of spontaneous physical activity [voluntary running (VR)] on metabolic syndrome-associated disorders, we used non-obese hereditary hypertriaclyglycerolemic (HHTg) rats as a unique model of dyslipidemia and metabolic syndrome. Various researchers have shown that forced and voluntary exercise produce very different effects on tested animals [48,49]. Animals forced to exercise experience physical and mental stress, so the physical activity in our experiments occurred under non-stressful conditions (VR on a rat wheel). Although ours was a non-obese model, because sucrose is associated with obesity, we also investigated whether the negative effects of sucrose consumption can be reduced by VR.

Our results suggest that VR, even in the absence of obesity, has a positive effect on lipid metabolism and reduces the negative effects of sucrose consumption. While separately VR and a high-sucrose diet had only a slight effect on total body weight, they had a significant effect on body composition and metabolism. Depending on the intensity of physical exercise, VR reduced the weight of perirenal and epididymal fat depots whilst also increasing lipolysis and protein content. Apart from these positive changes in adipose tissue, positive changes in the lipid metabolism of the VR rats resulted in lower concentrations of serum TAG and NEFA; these reductions were also correlated with VR intensity. Furthermore, the positive effect of VR on insulin sensitivity was confirmed by a higher ISI and by a reduced non-fasting insulin concentration. Collectively, these positive changes were associated with the following beneficial effects: lower sensitivity to oxidative stress; reduced lipid deposition in the liver, muscles, aorta and kidney; reduced content and changed composition of diacylglycerols (DAG) in the measured tissues; modified fatty acid composition in muscle membrane phospholipids; enhanced brown adipose tissue parameters.

It is believed that both fructose overfeeding and exercise lead to the increased production of reactive oxygen species (ROS) [50,51]. In agreement with this theory, we found increased levels of TBARS, a marker of lipoperoxidation, in both the plasma and tissue of the HSD rats. However, contrary to the theory, we also found that the TBARS levels actually decreased after VR. This may have been due to the adaptation of the organism to exercise-induced ROS production, which is associated with increased antioxidant capacity [52]. This idea is supported by the fact that we found increased antioxidant enzyme activity in the plasma and tissues of the VR rats. These results are in agreement with research which shows that exercise reduces oxidative stress [53].

One of the most serious complications associated with metabolic syndrome is increased lipid deposition in non-adipose tissues. Increased lipid deposition is associated with a variety of complications: in the liver, it leads to insulin resistance [15]; in the kidney, it can cause nephropathy [54]; in the heart and vessels, it may result in decreased ventricular distensibility and an increased risk of atherosclerosis [55,56]. Our results indicate that TAG content in the liver, kidney, heart and vessels can be significantly reduced by VR, even in the case of a high-sucrose diet. This could be due to the fact that the elevated energy expended during running leads to a higher utilization of tissue lipids and, consequently, to lower TAG content in both tissue and serum.

Increased amounts of lipids in muscles have been linked to muscle insulin resistance [13]. Our data demonstrate that VR substantially reduces the amount of TAG in gastrocnemius muscle and the diaphragm, and that such a reduction is quantitatively correlated with running intensity. Paradoxically, however, it has been shown that lipid content in the muscles of endurance-trained athletes increases even though their muscles are highly insulin sensitive [57,58]. This discrepancy is probably explained by the fact that the physical activity of endurance athletes is far more intense than that of VR rats. Nevertheless, the intensity displayed by our VR rats seems to be quite sufficient to have a significant effect in the treatment of dyslipidemia.

One of the mechanisms by which increased lipid content in the liver and muscles mediates insulin resistance is through the increased production of lipotoxic intermediates, primarily DAG and ceramides [16,17]. Hence, we determined the content of these intermediates in the liver, heart and soleus muscle.

DAGs are signal molecules capable of increasing the activity of some species of protein kinase C (PKC). Once activated, PKC phosphorylates the insulin receptor substrate (IRS), thereby inhibiting insulin signal transduction [59]. In our experiment, VR reduced the total content of DAG in the liver and heart of both dietary groups, as well as in the soleus of the HSD group. An important factor that could modulate the effect of DAG on insulin sensitivity is the composition of fatty acids, especially their saturation [60,61]. We found that VR significantly increases the proportion of polyunsaturated fatty acids in DAG, not only in skeletal muscle, as described by Bergman *et al*. [60], but also in the heart and liver, which has not previously been reported. However, it is not clear whether this effect is positive or negative because DAG subspecies consisting of polyunsaturated fatty acids have been described as more effective activators of PKCs *in vitro* [61]. Conversely, increased muscle DAG saturation is associated with insulin resistance and type 2 diabetes in human studies [62]. Furthermore, it can be assumed that intracellular localization and the position of individual DAG fatty acids may also be important factors in determining insulin signal transduction.

Ceramides are signal molecules that inhibit insulin signal transduction in muscles via the inhibition of protein kinase B (PKB). They are also associated with inflammation, oxidative stress, mitochondrial dysfunction and other metabolic disturbances [63,64]. We found that a high-sucrose diet led to a slight increase in ceramide species content in the heart, liver and soleus. However, VR had almost no effect on ceramide content in the studied tissues, which does not accord with reports of the reductive effect of physical activity on muscle ceramide content. For example, Amati *et al*. reported that endurance-trained athletes had a lower muscle ceramide content than obese humans [65]. Such discrepancies may be due to our use of non-obese subjects or even to the relatively short timeframe of our experiment (4 weeks compared with 16 weeks [66]). On the other hand, our results are in agreement with the theory expounded by Galbo and Shulman, who have shown that it is DAG content (and not ceramide content) that is associated with metabolic syndrome and hepatic insulin resistance [67].

From the above, it seems that the total amount of lipids and lipotoxic intermediates in tissue may be a key factor in the development of insulin resistance and metabolic syndrome. However, our results, together with evidence from the literature [19], suggest that the fatty acid composition of individual lipid classes could be an equally important factor, especially in the case of membrane phospholipids. The fatty acid profile of membrane phospholipids, particularly their saturation, influences the physical properties of membrane function, such as fluidity, permeability, signal transduction and the anchoring of membrane-related proteins [20,68]. Previously, the effect of physical activity on the fatty acid profiles of muscle membranes has only been reported for healthy humans or animals [69,70]. We show the potentially positive effects of VR on muscle membrane phospholipids in a dyslipidemia and metabolic syndrome model. In rats in both dietary groups, VR substantially reduced the amount of palmitooleic acid and altered the content of certain n-6 polyunsaturated fatty acids in soleus muscle membrane phospholipids. In the same rats, the Δ-5 desaturase activity index increased. Both a high content of palmitooleic acid and low Δ-5 desaturase activity index have been reported as BMI-independent predictors of insulin resistance and metabolic syndrome [71,72].

The mechanisms described above suggest that physical activity positively affects lipid metabolism, but it also appears to have beneficial effects for brown adipose tissue (BAT) metabolism [23,24]. BAT is able to degrade large amounts of lipids and produce heat; increased BAT activity is associated with the prevention of metabolic syndrome, dyslipidemia and obesity [25,27]. However, there is disagreement about the causal relationship between physical exercise and BAT metabolic activity. According to one theory, the heat produced in muscles during physical exercise leads to reduced BAT activity [73,74]. A contrary theory suggests that physical exercise increases BAT metabolic activity via the sympathetic nervous system [23,24]. Both of these hypotheses have only been verified indirectly by observing structural changes to BAT and the different mRNA expression of selected genes after physical activity. We directly determined the effect of VR on BAT activity by the *ex vivo* oxidation of exogenous albumin-bound fatty acids. We found that VR increased BAT activity in the CD+VR group, but not in the HSD+VR group. Our results for CD+VR support the hypothesis that after physical exercise BAT metabolic activity increases through the sympathetic nervous system. However, the results for HSD+VR indicate that high-sucrose feeding could interfere with this positive mechanism.

## Conclusion

Our results suggest that physical activity, even in the absence of obesity, can counteract the negative effects of sucrose consumption and the factors involved in the pathogenesis of metabolic syndrome-related disorders. In our study, voluntary running was associated with various beneficial changes, including lower concentrations of serum triacylglycerols and non-esterified fatty acids, reduced oxidative stress, and, in particular, decreased insulin resistance. All of these changes were to some extent connected to positive changes in lipid metabolism. Reduced lipid deposition in the liver, muscles, kidney and aorta was accompanied by the lower content and altered composition of diacylglycerols in the liver, heart and soleus muscle, as well as by fatty acid remodeling in muscle membrane phospholipids. We have suggested that the mechanism through which physical activity positively modulates lipid metabolism involves increased fatty acid oxidation in brown adipose tissue. These data provide important new findings about the positive effects of voluntary exercise and demonstrate its applicability to the treatment of dyslipidemia and metabolic syndrome.
